# Obesity mediates the relationship between depression and infertility: insights from the NHANES 2013-2018 cross-sectional study and a Mendelian randomization study

**DOI:** 10.3389/fendo.2024.1465105

**Published:** 2024-08-29

**Authors:** Ting Xu, Yuan Zhuang, Huabin Cao, Jingqi Yang

**Affiliations:** ^1^ Department of Ambulatory Surgical Center, Jiangxi Maternal and Child Health Hospital, Maternal and Child Health Hospital of Nanchang Medical College, Nanchang, China; ^2^ Department of Cardiovascular Medicine, Jiangxi Provincial People’s Hospital, The First Affiliated Hospital of Nanchang Medical College, Nanchang, Jiangxi, China

**Keywords:** depression, infertility, obesity, cross-sectional study, Mendelian randomization

## Abstract

**Background:**

Depression is increasingly recognized as a factor affecting infertility and the causal relationship between them remains controversial. The aim of this study was to explore the relationship between depression and infertility using Mendelian randomization (MR) and cross-sectional study, and to explore the potential mediating role of obesity.

**Methods:**

The cross-sectional study used data from the National Health and Nutrition Examination Survey (NHANES) 2013-2018. Multivariable-adjusted logistic regression was used to assess the association between depression and infertility risk, and mediation analysis was to examine the mediating effect of obesity. Then, we performed MR analyses to investigate the causal effect of depression on infertility. Instrumental variables for depression were obtained from a genome-wide association meta-analysis (135,458 cases and 344,901 controls), and summary level data for infertility were obtained from the FinnGen database (6,481 cases and 68,969 controls).

**Results:**

In the cross-sectional study, a total of 2,915 participants between the ages of 18 and 45 were included, of whom 389 were infertile. We observed that depression was strongly associated with an increased risk of infertility (OR=1.66, 95%CI: 1.19, 2.33), and this relationship remained significant in mild (OR=1.45, 95% CI: 1.09, 1.93), moderate (OR=1.89, 95% CI: 1.26, 2.84), and severe depression (OR=1.74, 95% CI: 1.02, 2.99). Mediation analysis showed that obesity mediated 7.15% and 15.91% of the relationship between depression and infertility for body mass index and waist circumference. Furthermore, depression significantly increased the risk of infertility in both the general obesity (OR=1.81, 95%CI=1.20-2.73, P<0.01) and abdominal obesity populations (OR=1.57, 95%CI=1.08-2.27, P=0.02) populations. In addition, the MR analysis also revealed a significant positive causal relationship between genetically predicted depression and infertility (OR=1.32, 95% CI: 1.03, 1.70).

**Conclusion:**

Depression is associated with an increased risk of infertility, with obesity playing a significant mediating role. This study underscores the importance of incorporating mental health and weight management in infertility treatment strategies.

## Introduction

The intricate tapestry of human reproduction is woven with both physiological and psychological threads, each playing a critical role in the successful conception and maintenance of pregnancy (1, 2). Among the myriad factors influencing fertility, depression has emerged as a significant contender, with a complex and often misunderstood relationship with infertility (3, 4). Depression, a psychiatric disorder with a profound impact on public health, is marked by a constellation of symptoms that include loss of interest, self-devaluation, and suicidal ideation (5). Its insidious effects extend beyond the individual, affecting social structures and exacerbating the escalating global disease burden (6). As the interplay between mental health and reproductive success becomes increasingly recognized, depression’s potential role in infertility has emerged as a critical research frontier (7).

Infertility, characterized by the inability to achieve a successful pregnancy after 12 months of regular, unprotected sexual intercourse, prevails as a growing concern (8). It affects an estimated 8-12% of couples globally, with female factors playing a significant role in a considerable proportion of these cases^8^. The emotional burden of infertility is well-documented, with depression often cited as a comorbid condition that may intensify the challenge of conception (9). Nevertheless, establishing a causal link between depression and infertility remains a complex endeavor, confounded by the interplay of various factors and the possibility of reverse causality.

Mendelian Randomization (MR), a powerful observational tool, offers a unique opportunity to assess causality by leveraging the random allocation of genes from parents to offspring (10). This approach mitigates many of the biases inherent in traditional observational research, providing a robust framework to investigate the potential causal effects of depression on infertility. The MR study by Ling et al. showed there were no evidence to support a causal or reverse causal relationship between depression and female reproductive disorders (11). In contrast, Zeng et al. provided evidence for a potential causal relationship between depression and female infertility, implicating a possible direct effect of depressive symptoms on the pathophysiology of infertility (12). It is also important to consider that in clinical practice, women with infertility are known to be at an increased risk for various psychological disorders, including depression (13). The contradictions among these studies highlight the necessity of employing a combination of MR and epidemiological approaches to elucidate these complex interactions and gain a more comprehensive understanding of the interplay between depression and female infertility.

We initially analyzed the relationship between depression and infertility through cross-sectional analysis in the NHANES database and explored whether obesity played a mediating role. Subsequently, we utilized investigate the causal relationship between depression and infertility using MR analysis. The aim of this study was to provide a comprehensive assessment of the relationship between depression and infertility using traditional epidemiological methods and the new insights provided by MR analyses. The findings may have significant implications for clinical practice, demonstrating the importance of including mental health as an integral part of infertility assessment and treatment.

## Methods

### Study design and population in the cross-sectional study

The National Health and Nutrition Examination Survey (NHANES) was utilized as the data source for the large cross-sectional study. NHANES is a program conducted by the National Center for Health Statistics (NCHS), Centers for Disease Control and Prevention (CDC), designed to assess the health and nutritional status of the civilian, non-institutionalized US population. The analysis included data from three consecutive two-year cycles spanning from 2013 to 2018, which allowed us to capture a robust sample representative of the US population’s health trends over time. The NHANES employs a stratified, multistage probability sampling design to ensure that the sample is representative of the population. NHANES relies on direct physical examination and clinical and laboratory tests, which are conducted in mobile examination centers (MECs) that travel nationwide. These MECs provide a standardized environment for health examinations, allowing for the collection of data that may be difficult or impossible to obtain through self-reports or healthcare provider records. Furthermore, NHANES employs personal interviews and related measurement procedures to gather information that encompasses more than just diagnosed conditions. It provides estimates on the prevalence of both diagnosed and undiagnosed diseases, including acute and chronic health conditions. The survey also assesses nutritional intake and status, chemical exposures, and a wealth of other health-related data.

The study protocol, with its rigorous sampling and data collection methods, was reviewed and approved by the NCHS Research Ethics Review Board. Informed consent was obtained from all participants, upholding the ethical standards of research with human subjects.

### Participant selection

**Figure 1:**
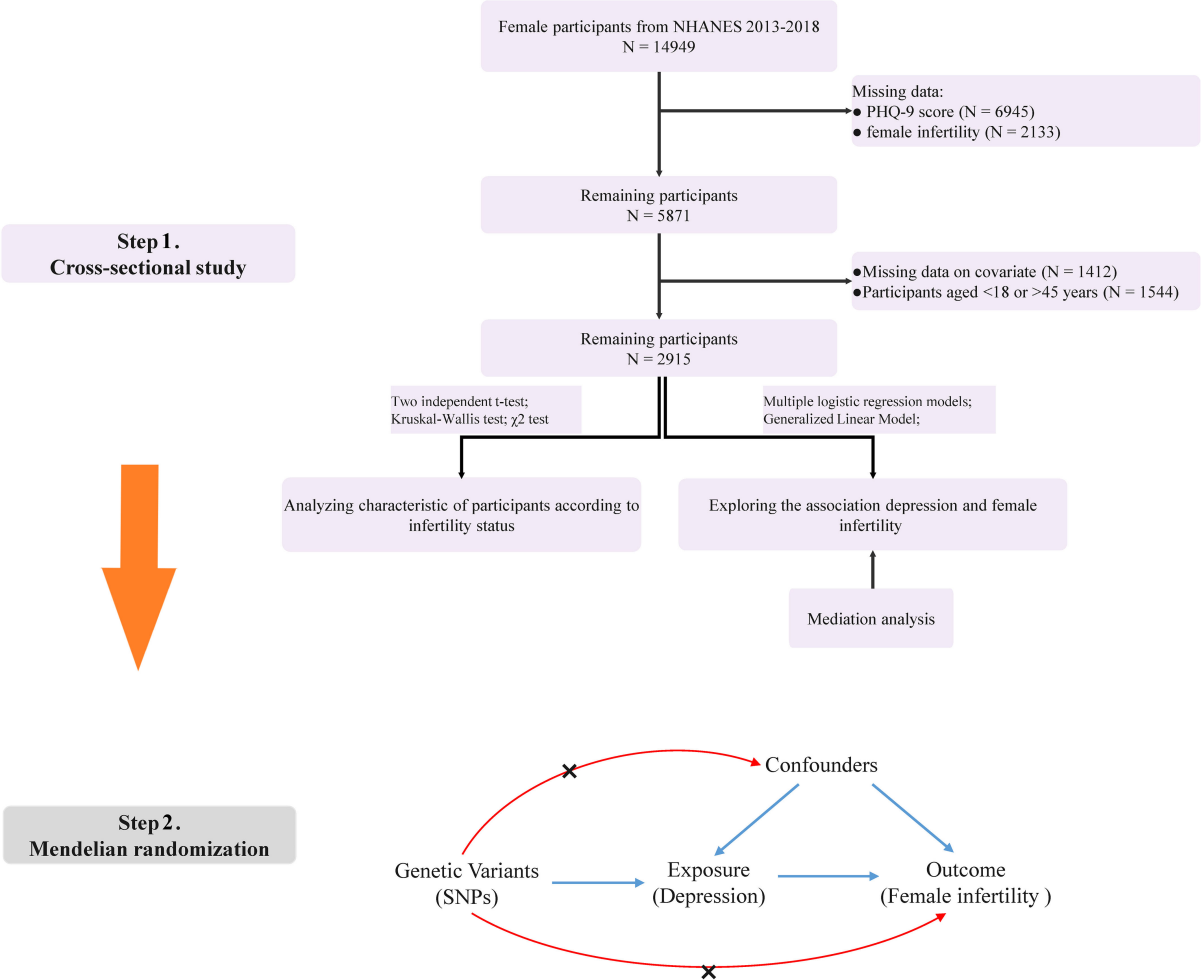
Flow chart of the study design.

The initial cohort was screened to exclude male individuals, those without Patient Health Questionnaire-9 (PHQ-9) score, individuals with incomplete data on female infertility, pregnant women, and cases with missing covariates. The final study population comprised 2915 participants aged 18-45 years old, as detailed in the flow diagram presented in **Figure 1**.

### Study variables

Depressive symptoms were quantified using the PHQ-9 instrument, a validated and reliable measure with a reported Cronbach’s α of 0.89 (14). The presence of clinically significant depressive symptoms was determined using the established threshold of a PHQ-9 score ≥ 10. For most analyses, participants were further categorized into no depression (PHQ-9 score: 0-4), mild (PHQ-9 score: 5-9), moderate (PHQ-9 score: 10-14) and severe depression (PHQ-9 score ≥15) (15). The choice of these specific categories was motivated by a dual rationale. Firstly, a pragmatic consideration was that the cut points of 5, 10, 15 and 20 are easy for clinical practitioners to remember and implement. The second reason was empiric, in that using different cut points did not noticeably change the associations between increasing PHQ-9 severity and measures of construct validity (16).

Infertility status was ascertained through responses to the Reproductive Health Questionnaire (RHQ), specifically questions RHQ-074: “Have you ever attempted to become pregnant over at least a year without becoming pregnant?” and RHQ-076: “Have you ever been to a doctor or other medical provider because you have been unable to become pregnant?”. Participants who answered “yes” to either of the two questions was considered as having infertility.

Obesity has a significant impact on the prevalence and clinical severity of diseases of the female reproductive system (17). Therefore, obesity, as a potential mediating factor, was assessed using Body Mass Index (BMI) and waist circumference (WC). BMI was calculated as weight in kilograms divided by the square of height in meters. WC was measured using a standardized protocol around the uppermost lateral border of the ilium while participants were standing. Individuals with BMI 18.5-22.4 kg/m^2^ and with WC < 80 cm were defined as the normal size. General obesity was defined as BMI ≥ 30 kg/m^2^ and abdominal obesity as WC ≥ 88cm according to the World Health Organization (WHO) definition of female obesity in the European population (18).

Covariates included sociodemographic factors including age, race, education level, marital status, and family poverty income ratio (PIR). Lifestyle factors such as smoking status and alcohol consumption were also considered. Female reproductive health factors included age at menarche, menstrual regularity in the preceding 12 months, history of pelvic infection, and oral contraceptive use.

### Data sources and single nucleotide polymorphism in exposure and outcome selection

The MR analysis was designed based on the following three basic assumptions (19): (1) the instrumental variable was strongly correlated with the exposure factor; (2) the instrumental variable was not associated with any potential confounders; (3) the instrumental variable was not directly related to the outcome, and its effect on the outcome was manifested only through the exposure. The two-sample MR analysis was used to assess the causal relationship between depression and infertility (**Figure 1**).

Genetic association estimates of single nucleotide polymorphisms (SNPs) with depression were obtained from the Psychiatric Genomics Consortium (PGC) (20). The PGC is recognized as a pioneering initiative in psychiatry, fostering collaboration and accelerating progress in unraveling the genetic basis of psychiatric disorders. We leveraged summary statistics from the PGC GWAS, which includes 7 samples (**Supplementary Table 1**): the PGC studies, deCODE, Generation Scotland, GERA (Genetic Epidemiology Research on Adult Health and Aging), iPSYCH, UK Biobank, and 23andMe (not publicly accessible) (20). The determination of depression cases was based on: structured diagnostic interviews, electronic medical records from healthcare institutions and self-reported clinical depression diagnoses or treatments, with diagnoses or treatments conducted by medical professionals (**Supplementary Table 1**). Participants (n = 135,458 cases, n = 344,901 controls) were of European genetic ancestry.

We employed a series of standard control strategies to identify valid IVs that meet the three core assumptions of MR. Initially, we selected SNPs that were randomly assigned and achieved a stringent threshold for depression (P < 5×10^-8^). However, due to the limited number of SNPs obtained, we adopted a relatively relaxed threshold, at the level of P < 5×10^-7^ (21). Linkage disequilibrium (LD) is a significant consideration in MR studies, reflecting the non-random association of alleles at nearby genetic loci. This proximity can lead to confounding in MR analyses if the instrumental variable, which is in LD with another variant influencing the outcome independently of the risk factor, is not properly accounted for (22). To mitigate this, we excluded SNPs with LD (R^2^<0.01, kb = 10000), where R^2^ was calculated using the formula R^2^ = (2 × EAF × (1 - EAF) × Beta^2^)/[(2 × EAF × (1 - EAF) × Beta^2^) + (2 × EAF × (1 - EAF) × N × SE^2^)] (23). In addition, to mitigate the influence of weak instrumental variables, we employed the F-statistic to evaluate the statistical robustness of the association between each SNP and the exposure variables. The F-statistic was calculated using the formula F = R²/(1 - R²) * (N - K - 1)/K. SNPs with an F-statistic value less than 10 were excluded, as an F-statistic value greater than 10 indicates sufficient strength to validate the reliability of the SNPs in our study. Ultimately, we identified a total of 12 independent genetic SNPs that were used as genetic instruments for depression (**Supplementary Table 2**).

Data for infertility were obtained from FinnGen database(https://www.finngen.fi). In FinnGen, female infertility was defined according to the International Classification of Diseases codes (ICD-10 N97, excluding N97.4; ICD9 628.0, 2-4, 8-9; ICD8 628). The GWAS summary statistics for infertility from FinnGen included 6,481 cases and 68,969 controls. To minimize possible bias due to population heterogeneity, all participants come from European.

### Statistical analysis

In the cross-sectional study, the data were analyzed using R software (version 4.3.0, http://www.R-project.org) and EmpowerStats software (version 4.1, http://www.empowerstats.net/analysis/). Continuous variables were evaluated using t-tests or Kruskal-Wallis tests, and categorical variables were examined using the chi-square test. The relationship between depression and infertility was investigated using multivariate logistic regression analysis with three progressively adjusted models: Model I (unadjusted), Model II (adjusted for age, race and marital status), and Model III (adjusted for all covariates). Penalized spline is a statistical technique used for smoothing data while allowing for the modeling of complex, non-linear relationships. This method incorporates a penalty term that controls the flexibility of the spline, preventing overfitting to the data and ensuring a more robust and interpretable model (24). Thus, the smoothed curve (penalized spline) analysis was employed to delineate the relationship between the depression score and the risk of infertility based on the fully adjusted model.

Having established the causal link between depression and endometriosis, we plan to further explore the role of obesity (including abdominal obesity and general obesity) in this association. We constructed a mediation effect model to analyze the impact of general obesity and abdominal obesity on the occurrence of endometriosis in individuals with depression. The mediation effect was quantified by calculating the mediation percentage (the ratio of the indirect effect to the total effect), and the significance of the mediation effect was tested using the Bootstrap resampling method (with the number of resamples set to 1000). A P-value of < 0.05 was considered to indicate a statistically significant difference.

In the MR analysis, the “TwoSampleMR” R package (version 0.5.6, https://github.com/MRCIEU/TwoSampleMR) was employed for the two-sample MR analysis, which aimed to delineate the causal relationship between depression and infertility. The analytical approach encompassed five distinct MR methods (25–29): inverse-variance weighted (IVW), MR-Egger regression, weighted median estimator (WME), simple mode, and weighted mode. The IVW method was selected as the primary analysis, with the Wald ratio calculated for each SNP to assess causality. To evaluate the potential for pleiotropy, MR-Egger regression and MR-PRESSO tests were implemented. A P-value threshold of P > 0.05 was applied to infer the absence of pleiotropic effects. The heterogeneity among the SNPs was assessed using Cochran’s Q-statistic. Where the p-value exceeded 0.05, indicating homogeneity, the results from the random-effects IVW model were considered. Conversely, in the presence of heterogeneity (P<0.05), a fixed-effect model was utilized. Furthermore, a sensitivity analysis was conducted using the leave-one-out method to ascertain the robustness of the MR estimates. This involved sequentially omitting individual SNPs and recalculating the effect sizes.

## Results

### Baseline characteristics of study participants in cross-sectional study

A total of 2,915 participants aged between 18 and 45 were enrolled, including 389 who were identified as infertile. The characteristics of the research participants were detailed in **Table 1** based on their infertile status. Self-reported infertility was significantly more prevalent among women who were older, had a higher PIR, were married, smokers, had a history of pelvic infection, used oral contraceptives, and exhibited higher WC and BMI (all P < 0.05).

**Table 1:** Baseline characteristics of the participants.

Characteristics	Non-Infertile (2526)	Infertile (389)	P-value
Age (Years)	32.36 ± 7.57	34.97 ± 6.97	<0.01
BMI (Kg/m^2^)	29.47 ± 8.26	32.19 ± 9.60	<0.01
Waist (cm)	95.45 ± 18.35	102.42 ± 20.28	<0.01
Race (%)			0.11
Mexican American	417 (16.51%)	60 (15.42%)	
Other Hispanic	255 (10.10%)	28 (7.20%)	
Non-Hispanic White	857 (33.93%)	156 (40.10%)	
Non-Hispanic Black	546 (21.62%)	83 (21.34%)	
Others	451 (17.85%)	62 (15.94%)	
Education (%)			0.68
Less than high school	374 (14.81%)	53 (13.62%)	
High school or equivalent	488 (19.32%)	71 (18.25%)	
College or above	1664 (65.87%)	265 (68.12%)	
PIR (%)			<0.01
<1	644 (25.49%)	83 (21.34%)	
>=1, <3	1081 (42.79%)	151 (38.82%)	
>=3	801 (31.71%)	155 (39.85%)	
Marital status (%)			<0.01
Married	1040 (41.17%)	231 (59.38%)	
Never married	829 (32.82%)	63 (16.20%)	
Others	657 (26.01%)	95 (24.42%)	
Drinking (%)			0.50
Never	636 (25.18%)	89 (22.88%)	
Every day or nearly every day	461 (18.25%)	62 (15.94%)	
3 to 4 times a week	353 (13.97%)	58 (14.91%)	
1 to 2 times a week	565 (22.37%)	99 (25.45%)	
Less than once a week	511 (20.23%)	81 (20.82%)	
Smoking (%)			0.02
Never	1798 (71.18%)	250 (64.27%)	
Now	457 (18.09%)	86 (22.11%)	
Former	271 (10.73%)	53 (13.62%)	
Menstrual Regularity (%)			0.54
No	260 (10.29%)	44 (11.31%)	
Yes	2266 (89.71%)	345 (88.69%)	
Age at Menarche (%)			0.45
< 13	1299 (51.43%)	208 (53.47%)	
≥ 13	1227 (48.57%)	181 (46.53%)	
PHQ-9 score	3.45 ± 4.25	4.51 ± 4.91	<0.01
Depression (%)			<0.01
No	2288 (90.58%)	330 (84.83%)	
Yes	238 (9.42%)	59 (15.17%)	
Pelvic infection (%)			<0.01
No	2413 (95.53%)	350 (89.97%)	
Yes	113 (4.47%)	39 (10.03%)	
Oral Contraceptive (%)			<0.01
No	845 (33.45%)	96 (24.68%)	
Yes	1681 (66.55%)	293 (75.32%)	

Data were presented as mean ± standard deviation or n(%). BMI, Body Mass Index; PIR, Poverty Impact Ratio; PHQ-9, Patient Health Questionnaire-9.

### Associations between depression and risk of infertile

**Figure 2:**
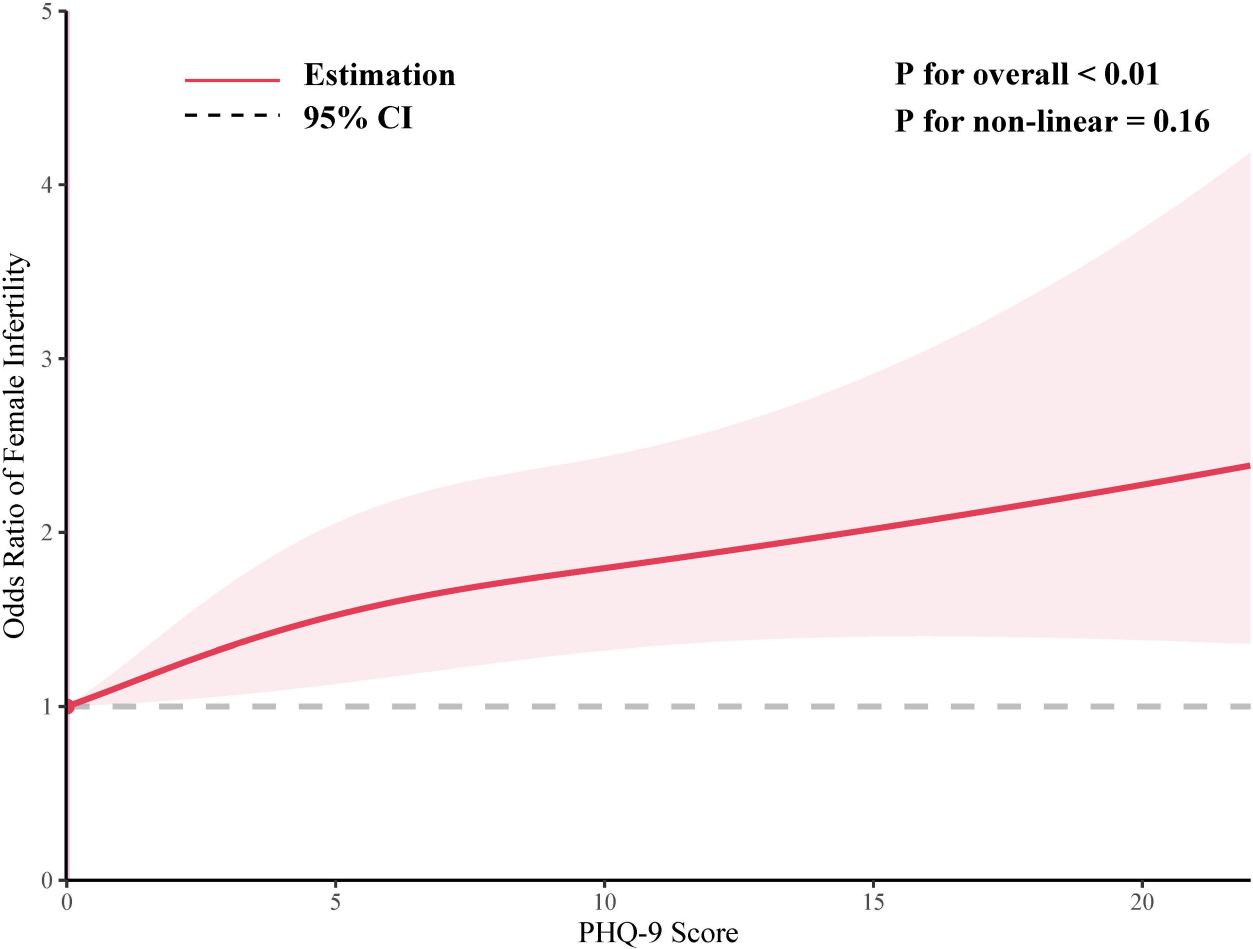
Smoothed curve analysis of PHQ-9 score for the estimation of the risk of infertility.

As shown in **Figure 2**, the smoothed curve analysis indicated a linear relationship between the PHQ-9 score and the risk of infertile. After adjusting for multiple variables, PHQ-9 score was statistically positively associated with risk of infertile (OR=1.05, 95%CI: 1.03, 1.08, **Table 2**) and compared to the female population without depression, those with depression exhibit a 66% increased risk of developing infertility (OR=1.66, 95%CI: 1.19, 2.33, **Table 2**). In addition, When the level of depression was categorized as mild, moderate or severe, all three groups had a significantly increased risk of infertility compared to the non-depressed group, with ORs (95% CIs) of 1.45 (1.09, 1.93), 1.89 (1.26, 2.84) and 1.74 (1.02, 2.99), respectively (**Table 2**).

**Table 2:** Association between depression and the risks of infertility.

	Non-adjusted	Adjust I	Adjust II
Depression			
No	1.0	1.0	1.0
Yes	1.72 (1.26, 2.34) <0.01	1.92 (1.39, 2.63) <0.01	1.66 (1.19, 2.33) <0.01
PHQ-9 Score	1.05 (1.03, 1.07) <0.01	1.06 (1.04, 1.09) <0.01	1.05 (1.03, 1.08) <0.01
PHQ-9 Score Category			
<5	1.0	1.0	1.0
≥5, <9	1.35 (1.03, 1.77) 0.03	1.47 (1.11, 1.94) <0.01	1.45 (1.09, 1.93) <0.01
≥10 <14	1.76 (1.20, 2.57) <0.01	2.05 (1.38, 3.04) <0.01	1.89 (1.26, 2.84) <0.01
≥15	1.99 (1.22, 3.24) <0.01	2.16 (1.31, 3.57) <0.01	1.74 (1.02, 2.99) 0.04

Non-adjusted model adjust for: None.

Adjust I model adjust for: Age; Race; Marital status.

Adjust II model adjust for: Age; Race; Education; Marital status; PIR; Drinking; Smoking; Age at Menarche; Menstrual Regularity; Oral Contraceptive; Pelvic infection.

### Mediating effect of obesity

**Figure 3:**
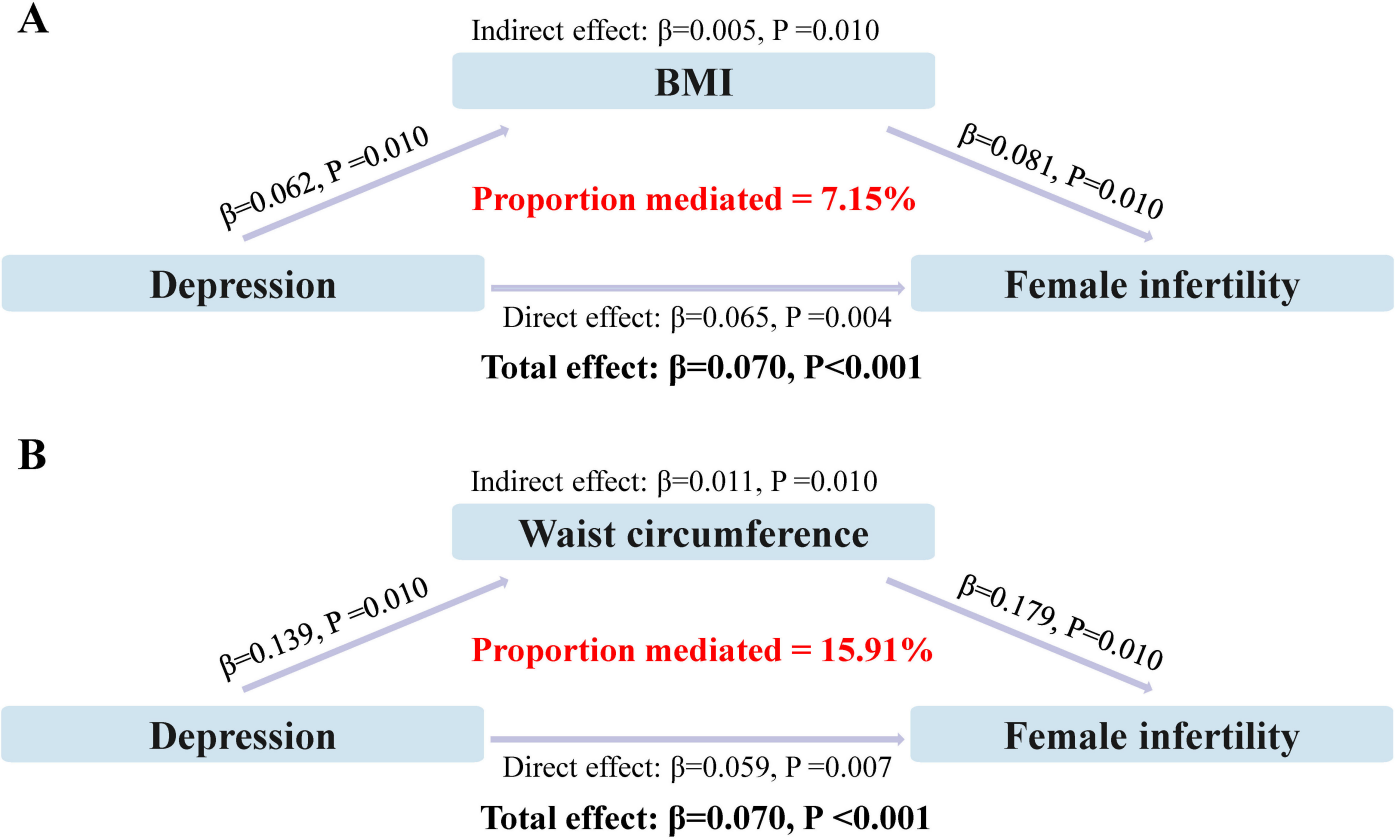
Analysis of obesity as a mediator between depression and infertility. **(A)** Mediating effect of BMI; **(B)** Mediating effect of waist circumference.

The direct and indirect effects of depression on infertility, mediated by indicators of obesity, are presented in **Figure 3**. The results of the study showed that both common measures of obesity (BMI and WC), significantly mediated the relationship between depression and infertility, with the mediating proportions of 7.15% and 15.91%, respectively (P<0.05).

### Relationship between depression and infertility in an obese population

We analyzed the relationship between depression and the risk of infertility in three separate groups: normal weight, general obesity, and abdominal obesity. Multivariate adjusted logistic analyses revealed that depression significantly increased the risk of infertility, both in the general obesity (OR=1.81, 95%CI: 1.20, 2.73, P<0.01, **Table 3**) and abdominal obesity populations (OR=1.57, 95%CI: 1.08, 2.27, P=0.02, **Table 3**). This relationship was not found in the normal-sized population (OR=1.52, 95%CI: 0.51, 4.56, P=0.45, **Table 3**).

**Table 3:** Association between depression and risk of infertility in different body sizes.

Body Shape	Depression	Non-adjusted	Adjust I	Adjust II
General obesity	No	1.0	1.0	1.0
	Yes	1.71 (1.18, 2.48) <0.01	2.00 (1.36, 2.93) <0.01	1.81 (1.20, 2.73) <0.01
	PHQ-9 Score	1.04 (1.02, 1.07) <0.01	1.06 (1.03, 1.09) <0.01	1.05 (1.02, 1.08) <0.01
Abdominal obesity	No	1.0	1.0	1.0
	Yes	1.56 (1.12, 2.19) <0.01	1.78 (1.25, 2.52) <0.01	1.57 (1.08, 2.27) 0.02
	PHQ-9 Score	1.04 (1.02, 1.07) <0.01	1.06 (1.03, 1.08) <0.01	1.05 (1.02, 1.08) <0.01
Normal Weight	No	1.0	1.0	1.0
	Yes	1.86 (0.74, 4.65) 0.18	2.16 (0.80, 5.81) 0.13	1.52 (0.51, 4.56) 0.45
	PHQ-9 Score	1.07 (1.00, 1.14) 0.04	1.08 (1.01, 1.15) 0.02	1.05 (0.98, 1.13) 0.16

Non-adjusted model adjust for: None.

Adjust I model adjust for: Age; Race; Marital status.

Adjust II model adjust for: Age; Race; Education; Marital status; PIR; Drinking; Smoking; Age at Menarche; Menstrual Regularity; Oral Contraceptive; Pelvic infection.

### Causal effects of depression on infertility in MR analysis

**Figure 4:**
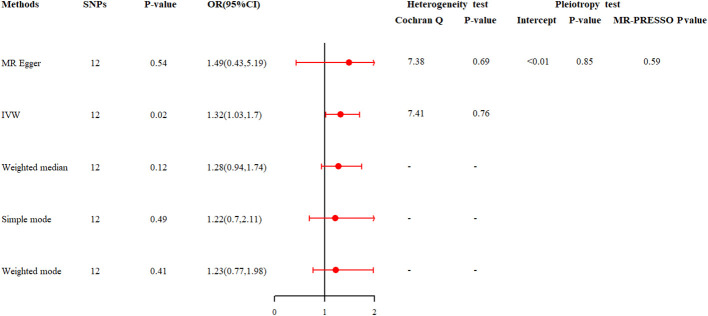
Summary of the MR analysis between depression and infertility.

MR analysis was then used to investigate the potential causal relationship between depression and infertility. In the two-sample MR analysis, 12 SNPs were extracted with depression as the exposure and infertility as the outcome. No significant heterogeneity was found in Cochran’s Q test (P>0.05) and a fixed effects model was used. The result of IVW analysis was found that a significant positive causal relationship between genetically predicted depression and infertility (OR=1.32, 95%CI: 1.03, 1.70, P=0.02) (**Figure 4**; **Supplementary Figure 1**). All alternative analysis methods yielded directional amplitudes consistent with the IVW analysis. MR-Egger regression (P = 0.69) and MR-PRESSO global test (P = 0.59) showed no significant horizontal pleiotropy (**Figure 4**). The robustness of results was confirmed by the leave-one-out sensitivity test (**Supplementary Figure 2**). In addition, when infertility was used as an exposure instrument and depression as an outcome instrument, the potential causal relationship between them was not observed in the MR analysis (OR = 1.04, 95% CI: 0.97, 1.11; P = 0.29, **Supplementary Table 3**).

## Discussion

The present study aimed to elucidate the causal relationship between depression and infertility, using both cross-sectional data and MR analysis. The cross-sectional study revealed that depression increases the risk of infertility. The two-sample MR analysis also confirmed a significant positive causal relationship between genetically predicted depression and infertility. Notably, we found an adverse effect of obesity in depression on the risk of female infertility. Our findings provide novel insights into the complex interplay between these two conditions and suggest that depression may indeed have a causal effect on infertility, as evidenced by both genetic and observational approaches.

We firstly analyzed the relationship between depression and the risk of infertility using cross-sectional data from NHANES 2013-2018. The results found that infertility was more prevalent among women with depression and that the risk of infertility was significantly higher for all levels of depression compared to those without depression. These results are also consistent with previous findings that the incidence of infertility was higher in patients with mood disorders (30, 31). Then, the fixed-effects model used in our MR analysis, justified by the absence of heterogeneity as indicated by Cochran’s Q test, yielded an odds ratio of 1.32 (95% CI: 1.03-1.70), suggesting a 32% increase in the odds of infertility per standard deviation increase in genetically predicted depression. This is similar to the results of the MR study by Cheng et al. (32) which confirmed a causal relationship between depression and infertility. However, the MR analysis by Ling et al. (11) did not find any evidence supporting a causal or reverse causal relationship between depression and infertility. It is important to note that in their MR study, the MR-PRESSO method yielded a P value of less than 0.05, indicating the presence of horizontal pleiotropy between exposure and outcome. This pleiotropy could potentially affect the reliability of the results. In addition, differences in study design, sample size, diagnostic criteria for depression and infertility, and the environments of the study populations, such as stress, lifestyle factors or socioeconomic status, may all influence the results of the research and the conclusions drawn.

In our analysis, we employed multiple MR methods to ensure the robustness of our findings. The IVW method, highlighted as our primary approach, is widely used due to its simplicity and effectiveness when the instrumental variables are assumed to be valid (25). The MR-Egger regression method was utilized to detect horizontal pleiotropy, a potential issue when the instrumental variables affect the outcome through pathways other than the exposure of interest (26). Additionally, we incorporated the Weighted Median Estimator, which is less sensitive to outliers and provides more robust estimates in the presence of weak instrumental variables or horizontal pleiotropy (27). The Simple Mode method, which considers the majority of the effect estimates, offers a more straightforward interpretation and is useful when the majority of the IVs are valid (27, 28). Lastly, the Weighted Mode method, which assigns weights based on the strength of the instruments, can be particularly advantageous when dealing with a set of instruments with varying levels of validity (29). We aim to conduct a comprehensive assessment of the causal relationship between depression and infertility, taking into account various potential sources of bias as well as the advantages of each method. By employing these different MR methods, although only the IVW method showed a P-value less than 0.05, the trends of the other four methods were consistent with the main results, indicating that the MR results are convincing.

Depression is increasingly recognized as contributing to an increased risk of infertility, and a complex interplay of factors may underlie this association. The specific mechanisms at play are multifaceted and may include the following: First, neuroendocrine disruptions associated with depression may directly affect reproductive function. The hypothalamic-pituitary-adrenal (HPA) axis, which is often dysregulated in people with depression, plays a critical role in regulating the menstrual cycle and gonadal hormone production (33). Hyperactivation of the HPA axis can result in elevated cortisol levels, which may interfere with the secretion of reproductive hormones, disrupting ovulation and potentially reducing fertility (34). Second, depression is associated with changes in levels of neurotransmitters such as serotonin and dopamine, which influence mood and behavior (35, 36). These neurotransmitters also regulate the hypothalamus, controlling the release of gonadotropin-releasing hormone (GnRH), a initiator of the reproductive cycle (37). Imbalances in these neurotransmitters may indirectly affect fertility. Finally, behavioral factors in individuals with depression, such as poor diet, sedentary lifestyle, smoking and alcohol consumption, can have a direct impact on fertility by affecting body weight, insulin sensitivity, and overall hormonal balance (38). These behaviors can have a direct impact on fertility by affecting body weight, insulin sensitivity and overall hormonal balance.

The mediating effect of obesity, as indicated by both BMI and WC, in the relationship between depression and infertility, is a novel finding of our study. The mediating proportions of 7.15% and 15.91%, respectively, suggest that obesity may partially account for the observed association between depression and infertility. Therefore, we also analyzed this in an obese population and found that depression significantly increased the risk of infertility in both general and abdominal obesity. This is in line with the literature highlighting the role of obesity as a risk factor for both depression and infertility (39).

In addition to the effects of depression, obesity increases the risk of infertility through multiple mechanisms. The excess adipose tissue in obesity induces hormonal perturbations, in particular escalating estrogen levels and decreasing sex hormone-binding globulin (SHBG), which can lead to menstrual irregularities and anovulation (40). At the same time, the chronic low-grade inflammation associated with obesity, adversely affects ovarian function and fallopian tube quality, making conception more difficult (41, 42). The psychological burden of obesity, particularly in the midst of depressive states, adds to the complexity. It can exacerbate depressive symptoms and increase cortisol levels, further disrupting the HPG axis (43). In addition, pharmacological treatment of depression, which can lead to weight gain, exacerbates obesity-related fertility problems (44). This interplay between physiological, psychological and iatrogenic factors provides a complex link between obesity and reduced fertility potential in people with depression.

Our findings have important clinical implications, suggesting that mental health screening and intervention should be an integral part of infertility evaluation and treatment. Given the high prevalence of depression and the significant mediating role of obesity, targeted interventions addressing both mental health and weight management could potentially improve fertility outcomes. It is crucial to acknowledge the limitations inherent in our study. Firstly, the cross-sectional design and MR analysis are subject to potential confounding factors. Although we adjusted for several covariates, the presence of residual confounders may still influence the study outcomes. Secondly, the conclusions drawn from the analysis are subject to the limitations inherent in the sample size of the cross-sectional study. The limited population size may affect the broader clinical interpretation and application of the study’s findings. Therefore, further larger-scale and more diverse prospective cohort studies are necessary to conduct to validate and extend these results. Thirdly, the reliance on self-reported data for infertility diagnoses could introduce reporting biases. Finally, the generalizability of our findings may be limited, as our study cohort was predominantly of US population. This limitation could restrict the applicability of our results to other ethnicities or global demographics.

In conclusion, our study advances the understanding of the complex interplay between depression, obesity, and infertility. The significant mediating effect of obesity and the elevated risk of infertility in obese populations with depression highlight the need for a comprehensive and integrated approach to managing reproductive health.

## Data Availability

The original contributions presented in the study are included in the article/**Supplementary Material**. Further inquiries can be directed to the corresponding authors.
