# Supplementary material for: Obesity mediates the relationship between depression and infertility: insights from the NHANES 2013-2018 cross-sectional study and a Mendelian randomization study
Source: Front Endocrinol (Lausanne). 2024 Aug 29;15:1465105. doi: 10.3389/fendo.2024.1465105 (PMC11394197; doi:10.3389/fendo.2024.1465105)
Supplement: Supplementary file 1 [file DataSheet1.docx]

Supplement Table 1. The general information of the seven depression cohorts

| **Sample** | **Country** | **Case ascertainment** | **Cases** | **Controls** |
| --- | --- | --- | --- | --- |
| PGC | Various | Structured diagnostic interviews | 16,823 | 25,632 |
| deCODE | Iceland | National inpatient electronic records | 1,980 | 9,536 |
| GenScotland | UK | Structured diagnostic interview | 997 | 6,358 |
| GERA | USA | Kaiser Permanente Northern California Healthcare electronic medical records (1995–2013) | 7,162 | 38,307 |
| iPSYCH | Denmark | National inpatient electronic records | 18,629 | 17,841 |
| UK Biobank (Pilot data release) | UK | From self-reported MDD symptoms or treatment or electronic records | 14,260 | 15,480 |
| 23andMeD (Discovery sample) | USA | Self-reported diagnosis or treatment for clinical depression by a medical professional | 75,607 | 231,747 |
| Total |  |  | 135,458 | 344,901 |

Supplementary Table 2. Single nucleotide polymorphisms used as instrumental variables for depression

| SNP | Chr | Position | Effect  allele | Other allele | Beta | Se | P-value | Sample  size | F-statistic |
| --- | --- | --- | --- | --- | --- | --- | --- | --- | --- |
| rs7531118 | 1 | 72837239 | C | T | 0.044997 | 0.008 | 2.15E-08 | 173005 | 31.6366 |
| rs12129573 | 1 | 73768366 | A | C | 0.047799 | 0.0082 | 5.45E-09 | 173005 | 33.9788 |
| rs2451828 | 5 | 7448796 | T | C | 0.1441 | 0.0273 | 1.30E-07 | 173005 | 27.8611 |
| rs12200766 | 6 | 95868745 | G | A | -0.0468 | 0.0091 | 3.10E-07 | 173005 | 26.4461 |
| rs6905391 | 6 | 28262686 | A | G | -0.074 | 0.0112 | 3.47E-11 | 173005 | 43.656 |
| rs56016904 | 9 | 5847764 | G | A | 0.051199 | 0.0099 | 2.55E-07 | 173005 | 26.7449 |
| rs58982057 | 9 | 11144237 | T | C | -0.0519 | 0.01 | 1.90E-07 | 173005 | 26.9399 |
| rs7856424 | 9 | 1.2E+08 | T | C | -0.045 | 0.0088 | 3.01E-07 | 173005 | 26.146 |
| rs10825942 | 10 | 58720316 | G | T | 0.044203 | 0.0082 | 7.46E-08 | 173005 | 29.0579 |
| rs12552 | 13 | 53625781 | G | A | -0.0405 | 0.008 | 3.90E-07 | 173005 | 25.627 |
| rs1950829 | 14 | 42097937 | G | A | -0.0454 | 0.0079 | 8.15E-09 | 173005 | 33.0308 |
| rs2060886 | 18 | 53167520 | C | T | 0.043398 | 0.008 | 5.76E-08 | 173005 | 29.4278 |

SNP: Single nucleotide polymorphisms; Chr: Chromosome; Se: Standard error.

Supplement Table 3. The reverse MR analysis between infertility and depression

| Exposure | Outcome | Method | SNP | β | P value | OR(95%CI) |
| --- | --- | --- | --- | --- | --- | --- |
| Infertility | Depression | MR Egger | 8 | -0.09 | 0.22 | 0.92(0.81-1.04) |
| Infertility | Depression | Weighted median | 8 | -0.001 | 0.94 | 0.99(0.92-1.08) |
| Infertility | Depression | Inverse variance weighted | 8 | 0.04 | 0.29 | 1.04(0.97-1.11) |
| Infertility | Depression | Simple mode | 8 | -0.01 | 0.9 | 0.99(0.88-1.12) |
| Infertility | Depression | Weighted mode | 8 | -0.01 | 0.86 | 0.99(0.89-1.10) |


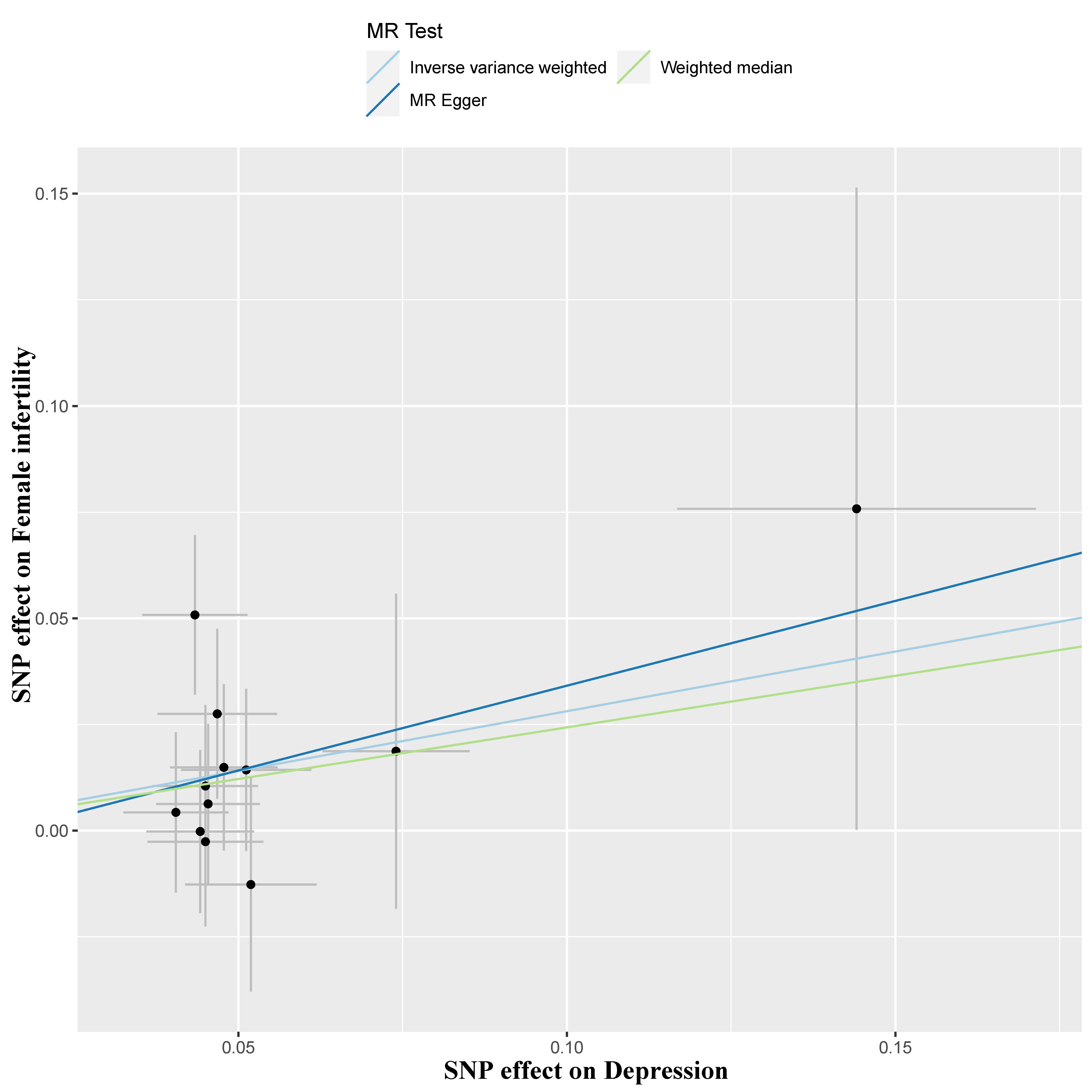


Supplement Figure 1. Scatter plot of genetic correlations of depression and female infertile using different MR methods.


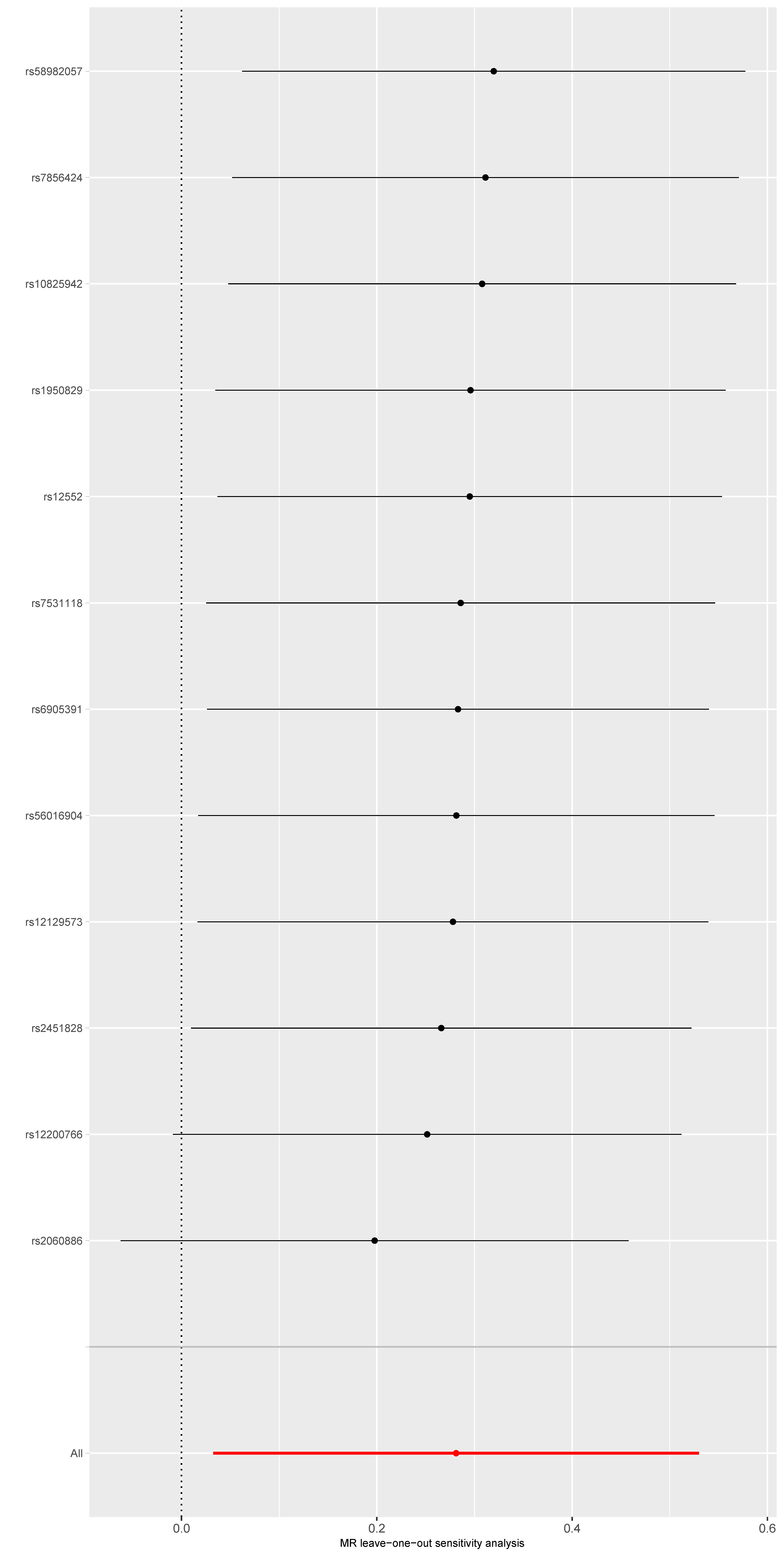


Supplement Figure 2. Forest plot of the causal effects of depression associated SNPs on infertility.
